# Families Enriched for Exceptional Longevity also have Increased Health-Span: Findings from the Long Life Family Study

**DOI:** 10.3389/fpubh.2013.00038

**Published:** 2013-09-30

**Authors:** Paola Sebastiani, Fangui X. Sun, Stacy L. Andersen, Joseph H. Lee, Mary K. Wojczynski, Jason L. Sanders, Anatoli Yashin, Anne B. Newman, Thomas T. Perls

**Affiliations:** ^1^Department of Biostatistics, Boston University School of Public Health, Boston, MA, USA; ^2^Geriatrics Division, Department of Medicine, Boston University School of Medicine and Boston Medical Center, Boston, MA, USA; ^3^Department of Epidemiology, Sergievsky Center, Taub Institute for Research on Alzheimer’s Disease and the Aging Brain, Columbia University, New York, NY, USA; ^4^Department of Genetics, Washington University, St. Louis, MO, USA; ^5^Department of Epidemiology, Graduate School of Public Health, University of Pittsburgh, Pittsburgh, PA, USA; ^6^Social Science Research Institute, Duke University, Durham, NC, USA

**Keywords:** health-span, longevity, onset of disease, survival analysis, Weibull regression

## Abstract

Hypothesizing that members of families enriched for longevity delay morbidity compared to population controls and approximate the health-span of centenarians, we compared the health-spans of older generation subjects of the Long Life Family Study (LLFS) to controls without family history of longevity and to centenarians of the New England Centenarian Study (NECS) using Bayesian parametric survival analysis. We estimated hazard ratios, the ages at which specific percentiles of subjects had onsets of diseases, and the gain of years of disease-free survival in the different cohorts compared to referent controls. Compared to controls, LLFS subjects had lower hazards for cancer, cardiovascular disease, severe dementia, diabetes, hypertension, osteoporosis, and stroke. The age at which 20% of the LLFS siblings and probands had one or more age-related diseases was approximately 10 years later than NECS controls. While female NECS controls generally delayed the onset of age-related diseases compared with males controls, these gender differences became much less in the older generation of the LLFS and disappeared amongst the centenarians of the NECS. The analyses demonstrate extended health-span in the older subjects of the LLFS and suggest that this aging cohort provides an important resource to discover genetic and environmental factors that promote prolonged health-span in addition to longer life-span.

## Introduction

The Long Life Family Study (LLFS) is an ongoing study of longevity and healthy aging in 583 families and almost 5000 family members demonstrating clustering for longevity. Median age of the probands and their siblings (the older generation or “generation one”) at enrollment was 92 years with an age range of 72–109 years. This substantial longevity was associated with decreased prevalence of age-related diseases such as diabetes and peripheral artery disease compared to subjects in the Framingham Heart Study and Cardiovascular Health Study (1).

However, disease prevalence may fail to capture important differences in health-span, that is, age of onset (2), particularly when comparing individuals with average life-span to individuals with extended life-span who may not escape morbidity but rather compress it toward the ends of their lives (3). Based on the hypothesis that age of onset of disease is a more informative metric of health-span than lifetime prevalence of disease, we set out to use survival analyses to study the health-span of LLFS generation one relative to population and spousal controls without familial longevity. In addition, we compare the health-span of LLFS generation one to the health-span of centenarians enrolled in the New England Centenarian Study (NECS) for whom we have demonstrated a substantial delay of age of onset of major age-related diseases, particularly for ages 105+ years (4).

## Materials and Methods

### Study subjects

The LLFS enrolled subjects between 2006 and 2009 via three American and one Danish field centers. Probands were screened using the Family Longevity Selection Score (FLoSS), which scored the degree of familial longevity using sex and birth-year cohort survival probabilities of the proband and their siblings (5). Eligibility of sibships for the study was based on a FLoSS score >7, and lack of cognitive impairment in the proband and at least one living sibling. The NECS has enrolled age-validated centenarians, their offspring and controls throughout the USA since 1994 (6). The NECS provided two comparison groups for this study: a control group of subjects enrolled because of either lack of longevity in their deceased parents (one parent died at the average life expectancy of their cohort and the other parent did not live beyond the age of 85 years) or because they were spouses of centenarian offspring, and centenarians and nonagenarian siblings of centenarians (Table 1). All participants underwent informed consent.

**Table 1:** **Characteristics of the subjects in the four groups**.

	LLFS probands and siblings	LLFS spouses	NECS nonagenarians and centenarians	NECS controls
Number of subjects	1493	192	1807	433
Number of families	583	192	1397	333
Age at enrollment^a^	92 (72–109)	85 (55–101)	100 (81–118)	72 (46–90)
Age last contact^a^	94 (72–110)	87 (58–102)	103 (87–119)	77 (47–96)
Birth-year cohort^b^	1916 (1898–1937)	1923 (1908–1953)	1900 (1880–1917)	1931 (1913–1957)
Follow up^a^	2 (0–6)	2 (0–4)	2 (0–13)	8 (0–10)
Male sex (% male)	709 (48)	42 (23)	456 (25)	234 (54)
Age last contact^a^ – males	93 (75–108)	89 (66–102)	102 (88–115)	77 (47–90)
Age last contact^a^ – females	95 (72–110)	87 (58–99)	103 (87–119)	77 (54–96)
Cancer (%)	26	22	19	32
COPD (%)	5	5	5	9
CVD (%)	44	37	48	27
Dementia (%)	9	5	31	4
Diabetes (%)	9	11	7	17
Hypertension (%)	54	62	43	61
Osteoporosis (%)	41	39	28	27
Parkinson’s (%)	1	1	2	2
Stroke (%)	19	15	19	11

The table reports summary demographic and health profiles in terms of prevalence of age-related diseases at last contact.

^a^Median age (range) in years.

^b^Median year (range).

### Data

In both LLFS and NECS, socio-demographic, vital status, and medical history data were collected via mailed questionnaires or in-person visits. Medical history questionnaires in both studies asked similar questions. Next-of-kin assisted when necessary. Ages of onset for the following diseases were used for the analysis: cancer (excluding non-melanoma skin cancers), chronic obstructive pulmonary disease (COPD: emphysema, bronchiectasis, and/or chronic bronchitis), cardiovascular disease (CVD: myocardial infarction, angina pectoris, congestive heart failure, atrial fibrillation, and/or valvular heart disease), dementia, diabetes, hypertension, osteoporosis (as diagnosed, or reported hip, wrist, and/or vertebral fracture at age 50 and older), Parkinson’s disease, or stroke. Because dementia was based on self- or proxy-reported medical history with possibly low sensitivity, a report of dementia should be considered as “severe dementia” rather than mild or moderate cognitive impairment. The selected diseases comprise the top 10 most prevalent diseases to afflict people age 65+ years according to the United States Centers for Disease Control (CDC) (7). In the NECS we compared subject’s (or their proxy’s) responses to the medical history questionnaire with their medical records for a random subsample of 90 subjects and for the diseases included in the analysis reported here the agreement was 100% (4). Overall morbidity was defined as one or more of the following: cancer, COPD, CVD, dementia, diabetes, or stroke. As in (4), hypertension was not included in the definition of overall morbidity because, if present, it was being treated with medication, thus markedly decreasing its effect upon morbidity. In both the NECS and LLFS, annual follow ups are conducted over the phone to update information on vital status and changes in health status and medications. The medical history data at enrollment and data from annual follow-ups were merged to calculate age of onset of disease based on the age at first occurrence. In both LLFS and NECS, follow up data were updated to November, 2012.

### Statistical methods

**Figure 1:**
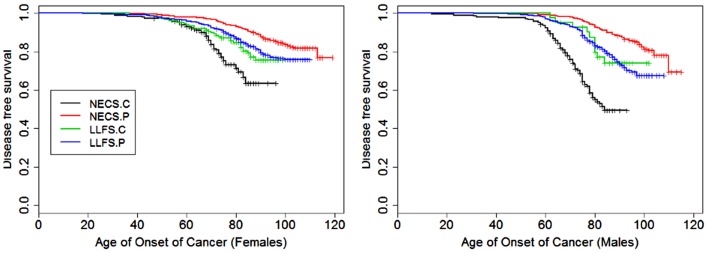
**Kaplan–Meier curves of survival free of cancer in NECS controls (NECS.C, black line), LLFS controls (LLFS.C, green line), LLFS probands and siblings (LLFS.P, blue line), and NECS centenarians (NECS.P, red line)**. Left panel: females; right panel: males. Skin cancer was not included.

**Figure 2:**
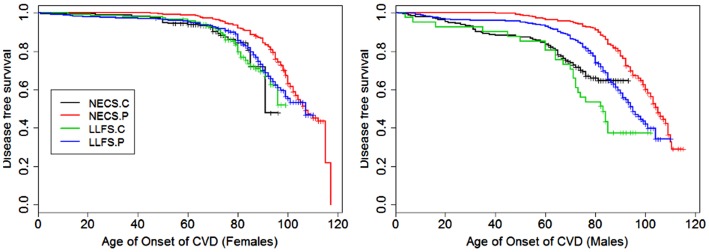
**Kaplan–Meier curves of survival free of cardiovascular disease (CVD) in NECS controls (NECS.C, black line), LLFS controls (LLFS.C, green line), LLFS probands and siblings (LLFS.P, blue line), and NECS centenarians (NECS.P, red line)**. CVD definition included myocardial infarction, angina pectoris, congestive heart failure, atrial fibrillation, and/or valvular heart disease. Left panel: females; right panel: males.

**Figure 3:**
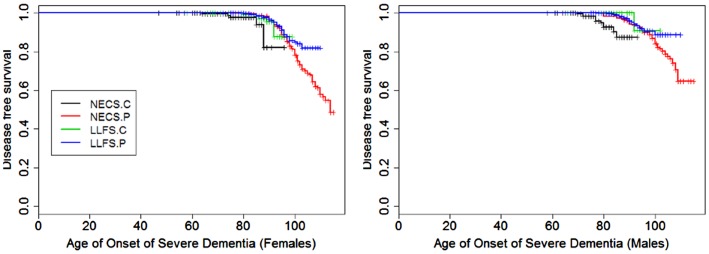
**Kaplan–Meier curves of survival free of severe dementia in NECS controls (NECS.C, black line), LLFS controls (LLFS.C, green line), LLFS probands and siblings (LLFS.P, blue line), and NECS centenarians (NECS.P, red line)**. Left panel: females; right panel: males.

**Figure 4:**
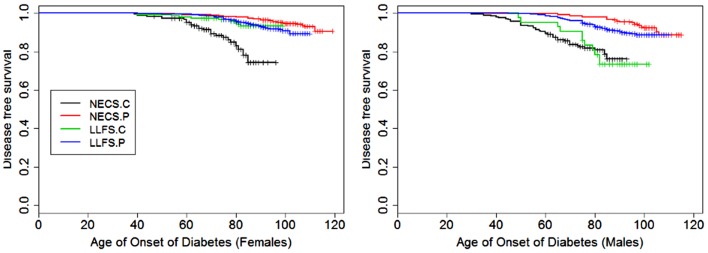
**Kaplan–Meier curves of survival free of diabetes in NECS controls (NECS.C, black line), LLFS controls (LLFS.C, green line), LLFS probands and siblings (LLFS.P, blue line), and NECS centenarians (NECS.P, red line)**. Left panel: females; right panel: males.

**Figure 5:**
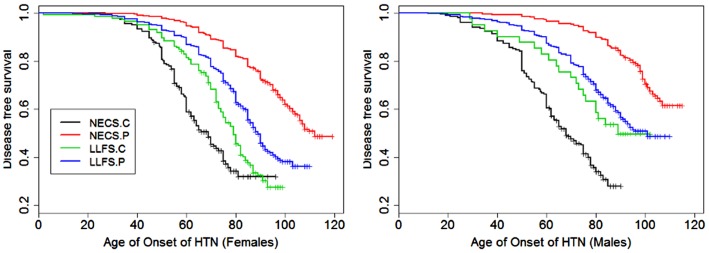
**Kaplan–Meier curves of survival free of hypertension in NECS controls (NECS.C, black line), LLFS controls (LLFS.C, green line), LLFS probands and siblings (LLFS.P, blue line), and NECS centenarians (NECS.P, red line)**. Left panel: females; Right panel: males.

**Figure 6:**
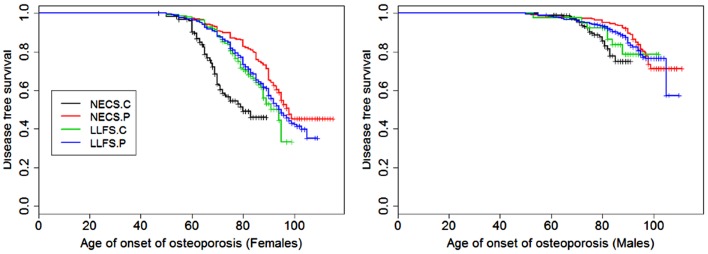
**Kaplan–Meier curves of survival free of osteoporosis in NECS controls (NECS.C, black line), LLFS controls (LLFS.C, green line), LLFS probands and siblings (LLFS.P, blue line), and NECS centenarians (NECS.P, red line)**. Osteoporosis age of onset was based on the earliest diagnosis or reported hip, wrist, and/or vertebral fracture at age 50 and older. Left panel: females; right panel: males.

**Figure 7:**
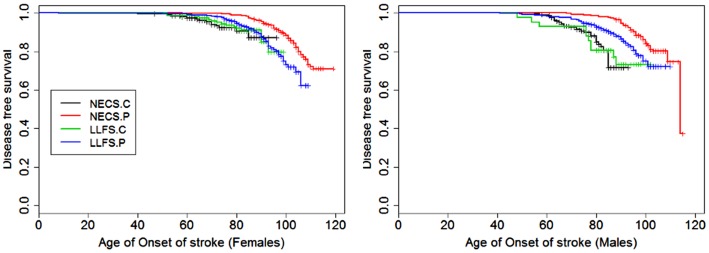
**Kaplan–Meier curves of survival free of stroke in NECS controls (NECS.C, black line), LLFS controls (LLFS.C, green line), LLFS probands and siblings (LLFS.P, blue line), and NECS centenarians (NECS.P, red line)**. Left panel: females; right panel: males.

**Figure 8:**
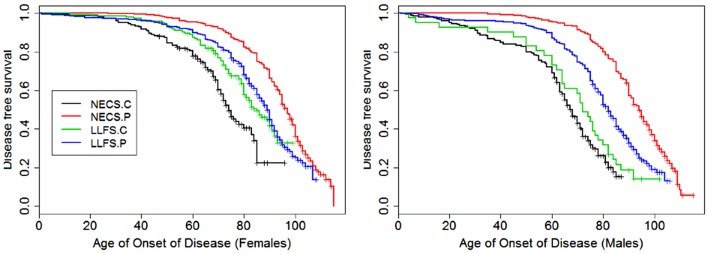
**Kaplan–Meier curves of survival free of morbidity in NECS controls (NECS.C, black line), LLFS controls (LLFS.C, green line), LLFS probands and siblings (LLFS.P, blue line), and NECS centenarians (NECS.P, red line)**. Morbidity was defined as one or more of the following: cancer, COPD, CVD, dementia, diabetes, or stroke. Left panel: females; right panel: males.

**Figure 9:**
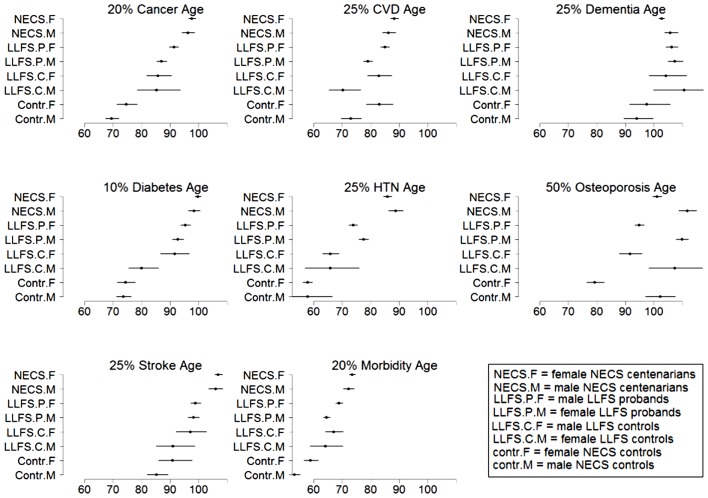
**Estimates and 95% credible intervals of the age at which *p*% of subjects in the various groups had onset of disease**. The inset describes the groups’ labels. Rationale for the choice of percentages *p* is in methods.

Patients’ characteristics are displayed by median, range, and proportions (Table 1). Disease-free survival stratified by gender and sample cohort is described by Kaplan–Meier curves (Figures 1–8). Last age of contact or age at death was used for censoring. Significant differences in hazard rates among groups and gender were tested using Bayesian parametric survival analysis with Weibull regression (4). Within sibship correlation was modeled using a normally distributed random effect per family in the log-hazard function (8). Comparisons between LLFS probands and siblings and their spouses relative to NECS centenarians and controls were summarized by hazard ratios (HRs) (Table 2), by the age estimated to reach specific percentiles of survival (Figure 9), and by the gains of each disease-free survival in years, compared to NECS controls (Table 3). The percentiles of survival for each disease are shown in Table 3 and were chosen to approximate the reported prevalence for each disease amongst older adults. Specifically, 20% prevalence for cancer for ages 65–80 was based on CDC Wonder^1^ (year 2009); 25% prevalence CVD for ages 65 and older was based on MMWR^2^; 25% prevalence for dementia was based on average of prevalence for age groups 65 and older and 85 and older reported by the Alzheimer’s organization in 2011^3^; 10% prevalence diabetes was based on CDC reported national statistics in 2011^4^; 25% prevalence hypertension was based on reference (9); 50% prevalence of osteoporosis was based on NHANES data^5^; and 25% stroke was based on data reported in (10).

**Table 2:** **Sex-specific hazard ratios of prevalent age-related diseases in subjects of the LLFS and NECS centenarians compared to NECS controls**.

Disease	Cont.F vs. Cont.M	LLFS.C.M vs. Cont.M	LLFS.C.F vs. Cont.F	LLFS.C.F vs. LLFS.C.M	LLFS.P.M vs. Cont.M	LLFS.P.F vs. Cont.F	LLFS.P.F vs. LLFS.P.M	NECS.M vs. Cont.M	NECS.F vs. Cont.F	NECS.F vs. NECS.M
Cancer	0.65 (0.43; 0.93)	0.27 (0.15; 0.48)	0.41 (0.27; 0.64)	0.97 (0.51; 1.88)	0.24 (0.19; 0.31)	0.28 (0.20; 0.39)	0.74 (0.62; 0.87)	0.13 (0.10; 0.17)	0.18 (0.14; 0.25)	0.91 (0.77; 1.10)
CVD	0.52 (0.35; 0.77)	1.21 (0.77; 1.86)	1.03 (0.72; 1.41)	0.44 (0.28; 0.74)	0.69 (0.54; 0.88)	0.93 (0.68; 1.23)	0.70 (0.62; 0.78)	0.44 (0.33; 0.57)	0.76 (0.56; 1.00)	0.90 (0.78; 1.04)
Dementia	0.68 (0.24; 1.85)	0.17 (0.06; 0.63)	0.47 (0.19; 1.29)	1.88 (0.49; 5.39)	0.23 (0.13; 0.46)	0.39 (0.19; 0.97)	1.13 (0.79; 1.62)	0.27 (0.15; 0.55)	0.57 (0.27; 1.41)	1.39 (1.07; 1.87)
Diabetes	0.91 (0.56; 1.48)	0.48 (0.24; 0.90)	0.16 (0.08; 0.32)	0.31 (0.15; 0.69)	0.13 (0.09; 0.19)	0.11 (0.07; 0.17)	0.78 (0.61; 1.00)	0.08 (0.05; 0.11)	0.08 (0.05; 0.12)	0.89 (0.71; 1.10)
HTN	1.00 (0.54; 1.85)	0.3 (0.15; 0.72)	0.56 (0.44; 0.71)	1.66 (1.05; 2.69)	0.27 (0.14; 0.51)	0.33 (0.28; 0.40)	1.24 (1.08; 1.42)	0.15 (0.08; 0.28)	0.17 (0.15; 0.20)	1.16 (0.99; 1.34)
Osteoporosis	4.44 (3.00; 6.48)	0.74 (0.41; 1.37)	0.43 (0.30; 0.61)	2.54 (1.41; 4.46)	0.65 (0.47; 0.89)	0.43 (030; 0.61)	2.38 (2.02; 2.78)	0.59 (0.42; 0.82)	0.24 (0.19; 0.31)	1.80 (1.49; 2.18)
Stroke	0.59 (0.29; 1.06)	0.58 (0.26; 1.14)	0.58 (0.29; 1.21)	0.59 (0.27; 1.32)	0.30 (0.21; 0.46)	0.49 (0.29; 0.92)	0.94 (0.74; 1.18)	0.16 (0.11; 0.25)	0.25 (0.15; 0.47)	0.93 (0.73; 1.17)
Morbidity	0.63 (0.48; 0.81)	0.42 (0.26; 0.70)	0.55 (0.40; 0.74)	0.81 (0.45; 1.32)	0.41 (0.34; 0.50)	0.48 (0.39; 0.60)	0.74 (0.65; 0.84)	0.24 (0.20; 0.30)	0.36 (0.29; 0.45)	0.93 (0.78; 1.07)

Hazard ratios and 95% credible intervals (in parenthesis) estimated using Weibull regression. Diseases are: CVD, cardiovascular disease; HTN, hypertension; morbidity, one or more of the following: cancer, COPD, CVD, dementia, diabetes, or stroke. Groups are Cont.F, NECS controls, females; Cont.M, NECS controls, males; LLFS.C.F, LLFS spouse controls, females; LLFS.C.M, LLFS spouse controls, males; LLFS.P.F, LLFS probands and siblings, females; LLFS. P.M, LLFA probands and siblings, males; NECS.F, NECS centenarians, females; NECS.M, NECS centenarians, males.

**Table 3:** **Sex-specific gain in years of disease-free survival of LLFS subjects and NECS centenarians relative to NECS controls**.

Disease	Cont.F vs. Cont.M	LLFS.C.M vs. Cont.M	LLFS.C.F vs. Cont.F	LLFS.P.M vs. Cont.M	LLFS.P.F vs. Cont.F	NECS.M vs. Cont.M	NECS.F vs. Cont.F
Cancer (*p* = 0.20)	5.0 (0.9; 9.6)	15.7 (8.5; 24.4)	11.1 (5.6; 16.9)	17.5 (14.4; 20.5)	16.7 (12.6; 20.4)	26.7 (23.3; 30.0)	23.02 (19.0; 26.6)
CVD (*p* = 0.25)	10.0 (3.9; 15.9)	−2.7 (−9.1; 4.4)	−0.14 (−6.5; 6.3)	6.0 (1.8; 9.8)	2.0 (−3.0; 6.8)	13.3 (9.0; 17.7)	5.2 (0.3; 10.0)
Dementia (*p* = 0.25)	3.4 (−5.4; 12.9)	16.4 (4.3; 27.2)	6.9 (−2.3; 15.5)	13.4 (7.3; 18.8)	8.8 (0.3; 15.3)	11.9 (5.6; 17.1)	5.2 (−3.3; 11.5)
Diabetes (*p* = 0.10)	0.78 (−3.3; 5.0)	6.4 (0.9; 12.9)	17.2 (10.7; 23.3)	19.1 (15.6; 22.4)	20.9 (17.0; 24.5)	24.7 (21.2; 28.0)	25.3 (21.6; 28.6)
HTN (*p* = 0.25)	−0.0 (−8.5; 7.4)	8.0 (4.6; 11.8)	8.0 (4.6; 11.5)	19.6 (10.8; 27.4)	16.0 (13.6; 18.2)	30.8 (21.7; 38.8)	28 (25.7; 30.2)
Osteoporosis (*p* = 0.50)	−22.8 (−28.9; −16.6)	5.2 (−5.4; 16.6)	12.3 (7.2; 17.3)	7.7 (2.1; 13.4)	15.6 (11.9; 19.0)	9.6 (3.6; 15.5)	21.8 (18.1; 25.2)
Stroke (*p* = 0.25)	5.5 (−0.6; 13.1)	5.7 (−1.4; 14.2)	6.1 (−2.2; 13.7)	12.9 (8.5; 16.7)	8.1 (1.0; 13.5)	20.7 (15.9; 24.7)	16.1 (9.1; 21.2)
Morbidity (*p* = 0.20)	5.6 (2.6; 8.8)	10.8 (4.3; 17.7)	8.1 (4.1; 12.3)	11.3 (9.0; 13.5)	10.0 (7.0; 12.8)	18.9 (16.1; 21.7)	14.6 (11.6; 17.3)

Years delay and 95% credible intervals (in parenthesis) to have a proportion *p* of the group affected with disease. The delays were estimated using Weibull regression and the formula age = (ln(1/*p*)/λ)^1/γ^ where λ and *v* are the parameters of the hazard function *h*(age) = λ*v*(age^γ−1^) (see Supplementary Material for details). The proportion *p* used for each disease is reported in column 1. Columns report gain in years of disease-free survival in female controls from the NECS relative to male controls (**Cont.F vs. Cont.M**); in male spousal controls from the LLFS relative to NECS male controls (**LLFS.C.M vs. Cont.M**); in female spousal controls from the LLFS relative to NECS female controls (**LLFS.C.F vs. Cont.F**); in male probands and siblings from the LLFS relative to NECS male controls (**LLFS.P.M vs. Cont.M**); in female probands and siblings from the LLFS relative to NECS female controls (**LLFS.P.F vs. Cont.F**); in male NECS centenarians relative to NECS male controls (**NECS.M vs. Cont.M**); in female NECS centenarians relative to NECS female controls (**NECS.F vs. Cont.F**).

Models with two-way interactions between gender and group were fitted and Bayesian estimates of the HRs, ages at which specific proportions of the different comparison groups were disease-free and gain of disease-free survival were computed using Markov Chain Monte Carlo (11), as in (4). Uninformative priors were assumed for all parameters, and at least 10,000 simulated values were used to estimate the parameters as the medians of the samples generated from the posterior distributions. Credible intervals were estimated using the 2.5 and 97.5 percentiles from the samples generated from the posterior distribution. Statistical significance of HRs was based on 95% credible intervals not containing 1, while statistical significance of gains of disease-free survival was based on 95% credible intervals not containing 0. The survival distributions estimated using Weibull regression were plotted together with the Kaplan–Meier curves to evaluate adequacy of model fitting by visual inspection (see Supplementary Material).

All analyses were conducted in OpenBugs^6^ and R 2.14.

## Results

Table 1 shows summary statistics of the 4 comparison groups included in the analysis and the prevalences of age-related diseases. The NECS centenarians included 120 supercentenarians (age at death ≥ 110 years) for most of whom we observed compression of morbidity (4).

Figures 1–8 show gender and disease-specific Kaplan–Meier curves for survival free of each of seven age-related diseases and overall morbidity free survival in the four groups. COPD and Parkinson’s disease were rare in long-lived individuals and were not included in these analyses (COPD prevalence was <5% and Parkinson’s disease prevalence was <2%). For each of the seven diseases and overall morbidity, the Kaplan–Meier curves show a consistent pattern of delayed age of onset in the order: NECS controls < LLFS controls < LLFS proband generation < NECS centenarians. An exception is severe dementia, where the age of onset tended to be delayed in LLFS probands and siblings relative to the other groups. An additional finding in Figures 1–8 is the effect of gender: amongst NECS controls, females consistently delayed the onset of disease compared to males. However, the delay of disease onset in females relative to males was more moderate in participants of the LLFS and practically disappeared amongst NECS centenarians. Exceptions were hypertension, delayed only in male NECS centenarians, and osteoporosis, delayed in males of all four groups.

Table 2 shows the HRs estimated by Bayesian–Weibull regression. Gender was a significant effect modifier of survival free of cancer, CVD, hypertension, osteoporosis, stroke, and overall morbidity but not severe dementia or diabetes. The effect of gender varied widely and these differences translated into varying years of disease-free survival (Table 3; Figure 9). We next summarize the results by disease.

### Cancer

Female NECS controls had decreased risk of cancer compared to male NECS controls (HR: 0.65, 95% CI: 0.43; 0.93). Similarly, female LLFS probands and siblings had decreased risk of cancer compared to male probands and siblings (HR: 0.74, 95% CI: 0.62; 0.87) but gender did not modify the risk for cancer in the other groups. The HR for cancer in female probands and siblings of the LLFS was 0.28 compared to female NECS controls (95% CI: 0.20; 0.39) and even smaller for male probands and siblings compared to male NECS controls (HR: 0.24, 95% CI: 0.19; 0.31). The HR for cancer of male NECS centenarians was 0.13 compared to male NECS controls (95% CI: 0.10; 0.17) and 0.18 for female NECS centenarians compared to female NECS controls (95% CI: 0.14; 0.25). Figure 9 shows that, by the age of approximately 70 years in males and 75 years in females, 20% of NECS controls had a cancer event. The age at which the same percentage of LLFS spousal controls had a cancer event was approximately 85 years, and the age was delayed to approximately 90 years in female LLFS probands and siblings and past 95 years in NECS centenarians. Table 3 shows the gain of years of cancer-free survival. In LLFS probands and siblings, the estimated age at which 20% of subjects had a cancer event was delayed by approximately 17 years compared to NECS controls for both males and females.

### Cardiovascular disease

In both the NECS and LLFS, female controls had lower risk of CVD compared to male controls (Table 2: NECS HR: 0.52, 95% CI: 0.35; 0.77; LLFS HR: 0.44, 95% CI: 0.28; 0.74). The gender difference decreased amongst LLFS probands and siblings and NECS centenarians (HR: 0.70, 95% CI: 0.62; 0.78) and became not significant in NECS centenarians. LLFS male probands and siblings had smaller risk of CVD only compared to male NECS controls (HR: 0.69, 95% CI: 0.54; 0.8) while the decrease in risk of female probands and siblings compared to NECS female controls did not reach statistical significance. NECS centenarians had smaller risk for CVD compared to NECS controls and the risk was substantially smaller for males (HR: 0.44, 95% CI: 0.33; 0.57) than females (HR: 0.76, 95% CI: 0.56; 1.00). The difference in HR for NECS centenarians and LLFS probands and siblings was not significant (note the overlapping interval estimates). The reduced hazard rates translated into a 2–6 year delay in the age at which 25% of LLFS probands and siblings had a CVD event (Table 3; Figure 9), compared to NECS controls. The gain in years of CVD free survival was larger in NECS centenarians than LLFS probands and siblings, but the difference was not statistically significant.

### Severe dementia

Gender did not significantly change the risk for severe dementia in NECS controls and LLFS subjects, while female NECS centenarians had higher risk for dementia than male NECS centenarians (HR: 1.39, 95% CI: 1.07; 1.87). LLFS subjects had lower risk of dementia compared to NECS controls (male HR: 0.23, 95% CI: 0.13; 0.46, female HR: 0.39, 95% CI: 0.19; 0.97). The risk was slightly lower than the risk of severe dementia in NECS centenarians relative to NECS controls. The lower risk for dementia of LLFS subjects relative to NECS controls translated into a gain of 8.8–13.4 years in age at which 25% of the LLFS subjects had severe dementia compared to NECS controls (Table 3; Figure 9).

### Diabetes

With the exception of LLFS spouses, gender did not significantly modify the risk for diabetes in any of the four groups. LLFS probands and siblings had a substantially smaller risks of diabetes relative to NECS controls (HR: 0.13 for males and HR: 0.11 for females). Compared to NECS controls, LLFS spouses had smaller risk of diabetes, and so did NECS centenarians (HR: 0.08) (Table 2). The estimated age at which 10% of the sample had history of diabetes was 73 years for NECS controls, between 80 and 90 years for LLFS spouses, 95 years for LLFS siblings and probands and 99 years for NECS centenarians (Figure 9). Table 3 shows that LLFS probands and siblings delayed the age at which 10% of the cohort had diabetes by about 20 years relative to NECS controls. The delay in NECS centenarians was 25 years.

### Hypertension

The risk for hypertension was significantly smaller in LLFS probands and siblings than NECS controls (male HR: 0.27, 95% CI: 0.14; 0.51, female HR: 0.33, 95% CI: 0.28; 0.40). NECS centenarians had similar change in risk compared to NECS controls: HR: 0.15 (95% CI: 0.08; 0.28) in male centenarians relative to male NECS controls and HR: 0.17 (95% CI: 0.15; 0.20) in female centenarians relative to female NECS controls. Female NECS centenarians also had greater risk for hypertension relative to male centenarians although the increase was only marginally significant (HR: 1.16, 95% CI: 0.99; 1.34). The risk for hypertension in LLFS female spouses was 0.56 times the risk in NECS controls (95% CI: 0.44; 0.71) and smaller in males (HR: 0.3, 95% CI: 0.15; 0.72). LLFS probands and siblings delayed the age at which 25% subjects had hypertension by about 18 years compared to controls from the NECS (Figure 9; Table 3). The reduced risk for hypertension in female NECS centenarians compared to male NECS centenarians translated into a 1.8 years delay of the age at which 25% subjects had hypertension but the gain was not significant (95% CI: −0.2; 5.8).

### Osteoporosis

This disease was more prevalent and occurred at earlier ages in females than males of all four groups (Figure 6). The regression analysis showed that females had significant higher risk than males in the four groups but the risk decreased within LLFS subjects (HR: 2.54 for LLFS spouses and HR: 2.38 for LLFS probands and siblings) and NECS centenarians (HR: 1.80) compared to NECS controls (HR: 4.4). However, the overlapping credible intervals show that the trend was not significant. LLFS and NECS subjects delayed the age at which 50% subjects had the disease by 8–9 years compared to male NECS controls and between 16 and 22 years compared to female NECS controls. Male NECS centenarians had a 13.5 years gain in the age at which 50% subjects had the disease compared to female NECS centenarians and the gain was significant (95% CI: −15.5; −11.5).

### Stroke

The HR for stroke in LLFS male probands and siblings compared to male NECS controls was 0.30 (95% CI: 0.21; 0.46) and 0.49 when compared to female NECS controls (95% CI: 0.29; 0.92). The risk was smaller in NECS male centenarians (HR: 0.16, 95% CI: 0.11; 0.25) relative to male NECS controls and relative to female NECS controls (HR: 0.25, 95% CI: 0.15; 0.47). The age at which 25% of controls had a stroke event was approximately 87 years for males and approximately 95 years for females, 98 years for LLFS probands and siblings and >105 years in NECS centenarians.

### Overall morbidity

The onset of morbidity was delayed in female NECS controls compared to male NECS controls, and in LLFS and NECS subjects compared to controls (Figure 8). However, gender differences in LLFS spouses (HR: 0.81, 95% CI: 0.45; 1.32) and NECS centenarians (HR: 0.93, 95% CI: 0.78; 1.07) were not significant. LLFS male probands and siblings had 0.41 times the risk of morbidity compared to male NECS controls (HR: 0.41, 95% CI: 0.34; 0.50) and 0.48 times the risk compared to female NECS controls (HR: 0.48, 95% CI: 0.39; 0.60). The risk for morbidity was even smaller in NECS centenarians. The age at which 20% LLFS probands and siblings experienced morbidity was delayed by 11.3 years relative to male NECS controls and 10 years relative to female NECS controls (Table 3).

## Discussion

The analysis showed that LLFS probands and siblings markedly delay the age of onset of major age-related diseases relative to NECS controls. The LLFS subjects’ overall disease-free survival was almost comparable to that of NECS centenarians with the exception of severe dementia where the LLFS subjects demonstrated an even greater delay. This finding could be due to the LLFS inclusion criterion that required the proband and at least one sibling to be able to provide their own consents at the time of enrollment. Pulmonary disease was much less prevalent in LLFS probands and siblings and NECS centenarians (5%) compared to NECS controls and this result supports the importance of pulmonary health for longevity and the fact that poor pulmonary function is a strong predictor of mortality (12, 13).

A similar pattern of delay in age of onset was observed for nearly all the age-related diseases included in these analyses. Perhaps members of these families age at slower rates and they have biological factors that impact upon both rate of aging and pathogeneses of age-related diseases. In general, women delay age-related diseases such as CVD and stroke by about 5–10 years compared to men (14). However, several centenarian studies have shown that this female advantage disappears at very old ages (15, 16). In this study of LLFS subjects, we similarly found that overall morbidity and disease-free survival became indistinguishable between males and females at the oldest ages. Exceptions were osteoporosis which was delayed in males compared to females in all groups, and hypertension. A generally accepted hypothesis for this convergence in disease rates is that men who develop age-related diseases at younger ages die, leaving behind a selected cohort of male survivors with age-related disease rates similar to aged females. However, these men are relatively rare at the oldest ages and amongst extremely old subjects aged 110+ years, the female to male ratio is 9 to 1 (4).

A surprising result of the analysis was the delay in onset of morbidity of the LLFS spouses. This delay could be due to the fact that at enrollment, the spouses have already achieved ages substantially beyond average life expectancy for their cohort. The spouses might also share environmental factors and life styles of LLFS probands and siblings that are conducive to increased health-span. Additional analyses that correlate environmental and genetic factors to the health-spans of these subjects will be necessary to understand this result (17).

This study aimed at comparing the health-spans of LLFS subjects relative to NECS controls and centenarians without trying to discover risk factors associated with the different patterns of survival. Future analyses that integrate genetic and non-genetic data will be necessary to discover factors that promote and extend health-span. The study described here establishes the LLFS as an invaluable resource to discover genetic and environmental factors that promote exceptional health-span and life-span.

### Limitations

A limitation of this study is that we relied upon self-report for ascertainment of the ages of onset of the various diseases. However for all of the diseases included in the analysis, there was a very strong correlation between these reports and the subjects’ medical records for a subsample of NECS subjects. Also, all participants were subjected to similar methods of data collection and therefore, comparisons between them should still yield valid results, at least relative to one another. The problem of likely decreased sensitivity associated with self-report for diseases such as dementia and stroke can be minimized in future analysis of LLFS data by relying upon direct functional and diagnostic measures that have been performed on all subjects. Unfortunately, these do not help us in determining age of onset for diseases that have already become clinically evident.

An additional limitation is that this analysis combines reported medical history with prospectively collected data about onset of diseases in highly selected subjects, so that the HRs comparing subjects selected for familial longevity to controls selected for lack of familial longevity may be influenced by a survivor effect. Although this limitation does not affect the comparative description of the health-span of the study subjects and provides evidence that the LLFS is a valuable resource for studies of healthy aging and longevity, the magnitude of the HRs may not be directly applicable to the general population. The study subjects are currently followed up and future analyses of the offspring cohort will provide more general results.

## Conflict of Interest Statement

The authors declare that the research was conducted in the absence of any commercial or financial relationships that could be construed as a potential conflict of interest.

## Supplementary Material

The Supplementary Material for this article can be found online at http://www.frontiersin.org/Epidemiology/10.3389/fpubh.2013.00038/abstract

## Footnotes

^1^http://wonder.cdc.gov/cancer.html

^2^http://www.cdc.gov/mmwr/preview/mmwrhtml/mm6040a1.htm

^3^http://www.alz.org/downloads/facts_figures_2011.pdf

^4^http://www.cdc.gov/diabetes/pubs/factsheet11/tables1_2.htm

^5^http://www.cdc.gov/nchs/data/nhanes/databriefs/osteoporosis.pdf

^6^http://mathstat.helsinki.fi/openbugs/Home.html
